# Cost-effectiveness of tiotropium versus omalizumab for uncontrolled allergic asthma in US

**DOI:** 10.1186/s12962-018-0089-8

**Published:** 2018-01-30

**Authors:** Zafar Zafari, Mohsen Sadatsafavi, J. Mark FitzGerald, for the Canadian Respiratory Research Network

**Affiliations:** 1 Mailman School of Public Health, Columbia University, New York, USA; 2 Collaboration for Outcomes Research and Evaluation, Faculty of Pharmaceutical Sciences, University of British Columbia, Vancouver, Canada; 3 Institute for Heart and Lung Health (IHLH), Faculty of Medicine, University of British Columbia, Vancouver, Canada; 4 Respiratory Medicine Division, Faculty of Medicine, University of British Columbia, Vancouver, Canada

**Keywords:** Tiotropium, Omalizumab, Uncontrolled asthma, Cost-effectiveness analysis, Decision analysis, Markov model

## Abstract

**Background:**

A significant minority of asthma patients remain uncontrolled despite the use of inhaled corticosteroids (ICS) and long-acting beta-agonists (LABA). A number of add-on therapies, including monoclonal antibodies (namely omalizumab) and more recently tiotropium bromide have been recommended for this subgroup of patients. The purpose of this study was to assess the cost-effectiveness of tiotropium versus omalizumab as add-on therapies to ICS + LABA for patients with uncontrolled allergic asthma.

**Methods:**

A probabilistic Markov model of asthma was created. Total costs (in 2013 US $) and health outcomes of three interventions including standard therapy (ICS + LABA), add-on therapy with tiotropium, and add-on therapy with omalizumab, were calculated over a 10-year time horizon. Future costs and quality-adjusted life years (QALYs) were discounted at the rate of 3%. Multiple sensitivity analyses were conducted. Cost-effectiveness was evaluated at willingness-to-pay value of $50,000.

**Results:**

The 10-year discounted costs and QALYs for standard therapy were $38,432 and 6.79, respectively. The corresponding values for add-on therapy with tiotropium and with omalizumab were $41,535 and 6.88, and $217,847 and 7.17, respectively. The incremental cost-effectiveness ratios (ICER) of add-on therapy with tiotropium versus standard therapy, and omalizumab versus tiotropium were $34,478/QALY, and $593,643/QALY, respectively. The model outcomes were most sensitive to the costs of omalizumab but were robust against other assumptions.

**Conclusions:**

Although omalizumab had the best health outcomes, add-on therapy with tiotropium was a cost-effective alternative to omalizumab and standard therapy for uncontrolled allergic asthma at willingness-to-pay of $50,000/QALY.

**Electronic supplementary material:**

The online version of this article (10.1186/s12962-018-0089-8) contains supplementary material, which is available to authorized users.

## Background

A significant minority of asthma patients remain uncontrolled despite using a combination of inhaled corticosteroids (ICS) and long-acting beta-agonists (LABA). The burden of such uncontrolled asthma on the patient and society is high [1–4]. A number of novel therapeutic interventions for this sub-group of asthma patients have been developed, with improvement in asthma symptoms and a reduction in exacerbations documented in clinical trials [5, 6]. The earliest example of such a targeted treatment is omalizumab. Omalizumab is an anti-IgE monoclonal antibody that has been approved for treating adults with 12 year and older with severe allergic asthma [7]. The addition of omalizumab to standard ICS + LABA therapy has been shown to have a positive effect on health outcomes and in particular a reduction in the rate of asthma exacerbations [8]. Recently, tiotropium bromide has been shown to reduce the risk of asthma exacerbations when added to combination therapy [9, 10] and has subsequently received regulatory approval as an add-on therapy for the treatment of uncontrolled asthma [6]. Both treatments have been recommended at level 5 in the GINA asthma treatment continuum [11].

Given the constrained healthcare resources, in addition to treatment effectiveness, the resources that are required for a treatment should also be considered to maximize population health. There have been a number of studies evaluating both the cost and effectiveness of add-on therapy with omalizumab versus standard therapy in different settings [3, 12–17]. Willson et al. have recently studied the cost-effectiveness of tiotropium compared with standard therapy in the UK [10, 18]. However, to the best of our knowledge, no single study has compared all the three alternatives in a unified framework. Without such a study, patients, clinicians, and policy makers do not have sufficient evidence on the choice of optimal therapy.

The aim of the present study was to use an evidence-informed modeling approach to assess the health and economic consequences of the three strategies of continuation of standard therapy, add-on therapy with tiotropium, and add-on therapy with omalizumab for the treatment of uncontrolled allergic asthma.

## Methods

### Model

**Fig. 1:**
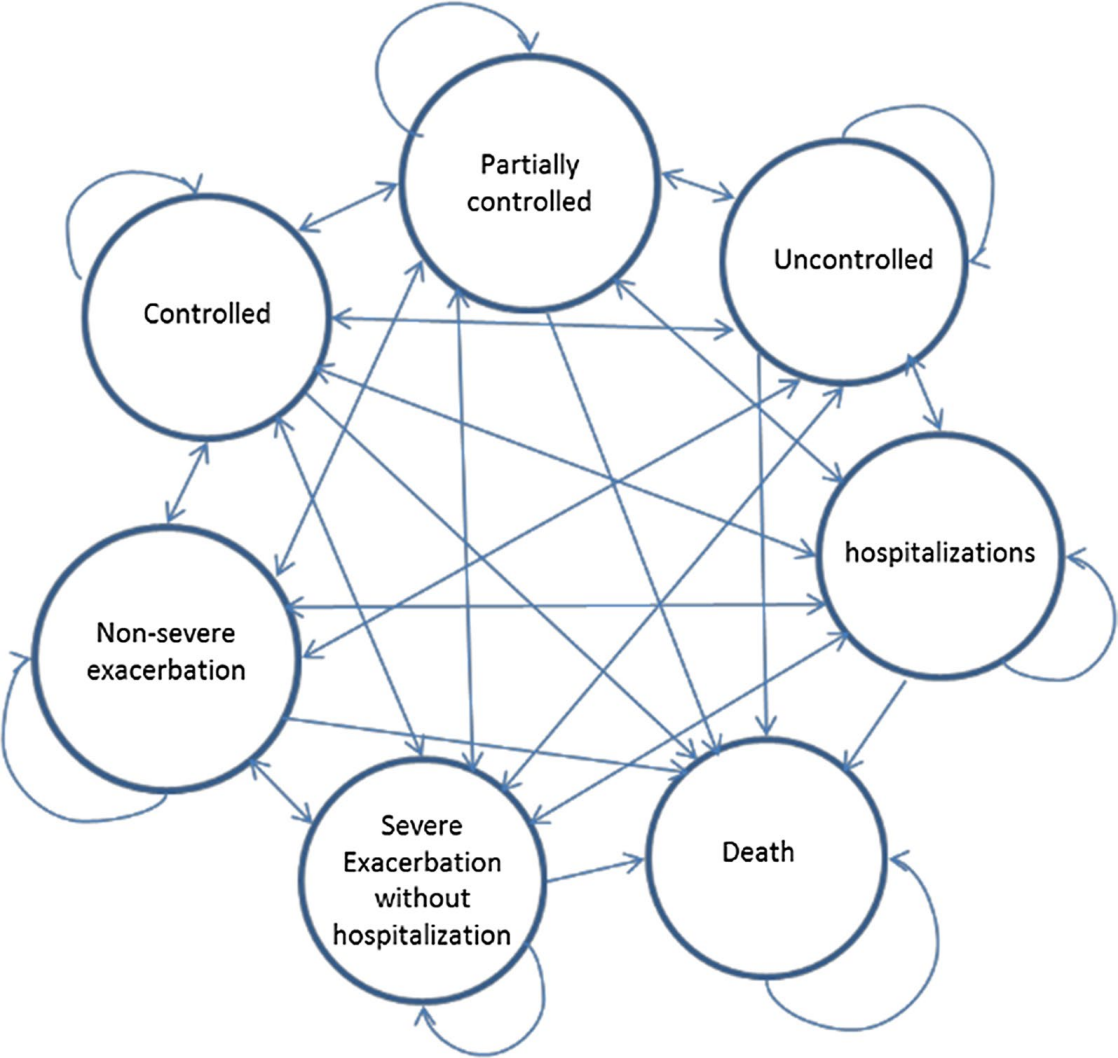
Markov model. States include controlled, partially controlled, uncontrolled, non-severe exacerbation, severe exacerbation without hospitalization, and hospitalization

We developed a probabilistic Markov model of asthma to project the costs and quality-adjusted life years (QALYs) of patient with uncontrolled allergic asthma under different treatments over 10 years. The setting for this evaluation is patients with severe allergic asthma in the US, and the study adopts a US societal perspective. As such, in the main analysis both direct and indirect medical costs were included. The choice of time horizon was in line with the similar cost-effectiveness studies in uncontrolled asthma [15]. Three interventions were modeled: continuation of standard therapy (high dose ICS + LABA), add-on therapy with tiotropium, and add-on therapy with omalizumab. The model consisted of seven health states including three asthma control states (controlled, partially controlled, uncontrolled), three exacerbation states (non-severe exacerbations, severe exacerbations without hospitalizations, and severe exacerbations requiring hospitalizations), and a death state. The cycle length of the study was 1 week. Previous studies that assessed the cost-effectiveness of omalizumab focused mainly on modelling the effect of treatment on transitioning between exacerbation free to exacerbation states [2, 3]. However, treatments can also result in improved symptom control. To enable a more comprehensive modeling of the treatment effects on both control and exacerbation states, we built our model structure in line with the previously published model of tiotropium, which modeled transition across both control levels and exacerbation states [10]. In particular, definition of heath states in our study were in line with the PrimoTinA-asthma trials [6] and the previously published cost-effectiveness study of tiotropium [10]. The control states represent the degree of severity of the daily symptoms of the disease. Non-severe exacerbations are defined as worsening of patient’s daily symptoms over and beyond the normal variations in the current level of symptom control. Severe exacerbations without hospitalizations represent a need for an oral corticosteroids use or doubling the doses of daily medications, and hospitalizations represent an episode of exacerbation that requires inpatient care [6]. Figure 1 represents the model structure. This probabilistic model enabled the projection of costs, QALYs, and total number of exacerbations for the three interventions under the study. All the future costs and QALYs were discounted at 3%.

### Parameters

Multiple outcome variables from published research were used to inform the model parameters, which are presented in Table 1. Model parameters were assigned probability distributions based on the degree of uncertainty reported by the original studies. The transition probabilities for moving between different health states for the standard therapy and add-on therapy with tiotropium were derived from a recent study by Willson et al. [18]. We could not find transition probabilities between the same health states for omalizumab in the published literature. Published research has mostly focused on the impact of omalizumab on the reduction of exacerbations as well as QALY gained [8, 19]. Thereby, using these data and the same methodology as in our earlier publications, we back-calculated the transition probabilities across different health states [4]. The details of this analysis are presented in the Additional file 1: Appendix 1.

**Table 1:** Model Parameters

Parameters	Value	Probability distribution
Baseline age	40	–
Background mortality rate [30]	US life tables	–
Monthly chance of death from hospitalization [31]	0.0248	Beta(1.099, 43.224)
Direct cost (2013-$US) [2, 3]		
Standard therapy costs (per person week)^a^		
Controlled	$46	–
Partially controlled	$47	–
Uncontrolled	$53	–
Treatment costs (per person week) [2, 3, 10, 21, 22]		
Tiotropium	$13	–
Omalizumab	$437	–
Cost of exacerbations (per person week) [2, 3]		
Non-severe exacerbation	$130	Gamma(100, 0.77)
Severe exacerbation without hospitalization	$594	Gamma(98.01, 0.17)
Hospitalization	$9900	Gamma(100.08, 0.01)
Indirect cost (per person week) (used only for sensitivity analysis) [1, 4, 22]		
Controlled	$165	–
Partially controlled	$185	–
Uncontrolled	$312	–
Exacerbation (Including not severe exacerbation, severe exacerbation and hospitalization)	$856	–
Health state utility values [10, 21, 22]		
Controlled	0.937	Beta(982.3883, 66.0517)
Partially controlled	0.907	Beta(378.5135, 38.8112)
Uncontrolled	0.728	Beta(1212.6010, 453.0598)
Non-severe exacerbation	0.649	Beta(1243.7040, 672.6349)
Severe exacerbation without hospitalization	0.570	Beta(1175.3160, 886.6418)
Hospitalization	0.330	Beta(613.7850, 1246.1690)

All costs are adjusted to 2013 US dollars using US consumer price index [22]

*Gamma(x, y)* distribution with shape parameter x, and rate parameter y, *Beta(x, y)* beta distribution with shape1 parameter x, and shape2 parameter y

^a^Details in the (Additional file 1: Appendix-1)

Treatment costs were derived from previously published US-based studies [2, 3]. We derived the costs of standard therapy from a study by Campbell et al. [3]. These costs have been estimated from samples with a mix of asthma control levels. We estimated the costs per levels of control based on the frequency of asthma control in the study sample and the evidence on the ratio of costs across control states [20]. The details of these calculations are shown in the Additional file 1: Appendix 2. The additional costs of omalizumab and tiotropium, as well as exacerbations costs were derived from the published literature [2, 3, 10, 21, 22]. All costs were adjusted to 2013 US dollars, details of which can be found in Table 1.

The health state utility values (utilities) were derived from a recently published study by Willson et al. [10]. The EQ-5D scores for the control states were estimated from the PrimoTinA-asthma trials and were converted into the utility values using the UK EuroQol tariff [10, 23]. The utility values for exacerbations states were derived from two recent studies by Willson et al. and LIoyd et al. [10, 24]. The details of these values and their probabilistic distributions are shown in Table 1.

### Analysis

We performed a main (base case) analysis by running the model with the point estimates of each parameter. Cost-effectiveness was interpreted around the reference willingness-to-pay (WTP) value of $50,000/QALY. A Monte-Carlo simulation with 10,000 runs was used to quantify uncertainty around the base case results. In each Monte Carlo run, a random sample was drawn from each distribution. From the results of the probabilistic analysis, we calculated 95% credible intervals (CrI) around point estimates of model outputs. We also generated the cost-effectiveness plane and the cost-effectiveness acceptability curve. The former is a scatterplot representing the joint distribution of the difference in costs and QALYs between the add-on therapies. The latter represents the possibility of each of the three treatments being cost-effective at different WTP values. The model was implemented in statistical programming language R (version 3.2.2) [25].

### Sensitivity analysis

To investigate the robustness of the outcomes with respect to changes in core model parameters, we performed a series of sensitivity analyses. The parameters evaluated were time horizon (changed to life time), baseline age (changed from 20 to 60 years), discount rate (0–5%), cost of omalizumab (changed by ± 25%), cost of tiotropium (changed by ± 25%), health state costs (changed by ± 25%), utilities (changed by ± 10%), and 30-day probability of death from severe asthma exacerbations (changed from 0.01 to 0.03).

## Results

Table 2 represents the results of the main analysis. For standard therapy, the total discounted 10-year costs and QALYs per person were $38,432 (95% CrI $32,075–$48,657), and 6.79 (95% CrI 6.63–6.96), respectively. The corresponding values for add-on therapy with tiotropium and with omalizumab were $41,535 (95% CrI $35,034–$54,699) and 6.88 (95% CrI 6.69–7.07), and $217,847 (95% CrI $214,477–$224,863) and 7.17 (95% CrI 6.99–7.37), respectively. The ICER of add-on therapy with tiotropium versus standard therapy was $34,478/QALY. The ICER of add-on therapy with omalizumab versus tiotropium was $593,643/QALY. The total number of exacerbations over 10 years per person was 33 for standard therapy, 24 for tiotropium and 18 for omalizumab.

**Table 2:** The expected values and 95% CrI of model outcomes over 10 years

Outcome	Standard therapy	Tiotropium	Omalizumab
Cost (95% CrI)	$38,432 ($32,075–$48,657)	$41,535 ($35,034–$54,699)	$217,847 ($214,477–$224,863)
QALYs (95% CrI)	6.79 (6.63–6.96)	6.88 (6.69–7.07)	7.17 (6.99–7.37)
Number of weeks with non-severe exacerbations (95% CrI)	20.04 (15.90–24.64)	14.53 (11.07–18.87)	9.92 (7.34–12.91)
Number of weeks with severe exacerbations without hospitalization (95% CrI)	11.39 (7.21–16.16)	8.22 (5.03–11.97)	7.15 (4.14–10.94)
Number of hospitalizations (95% CrI)	1.10 (0.45–2.19)	1.05 (0.31–2.46)	0.54 (0.14–1.48)
ICER			
*Tiotropium versus standard therapy*	Reference	$34,478/QALY	–
*Omalizumab versus tiotropium*	–	Reference	$593,643/QALY
*Omalizumab versus standard therapy*	Reference	–	$463,605/QALY

*CrI* credible interval, *QALY* quality-adjusted life year, *ICER* Incremental cost-effectiveness ratio

**Fig. 2:**
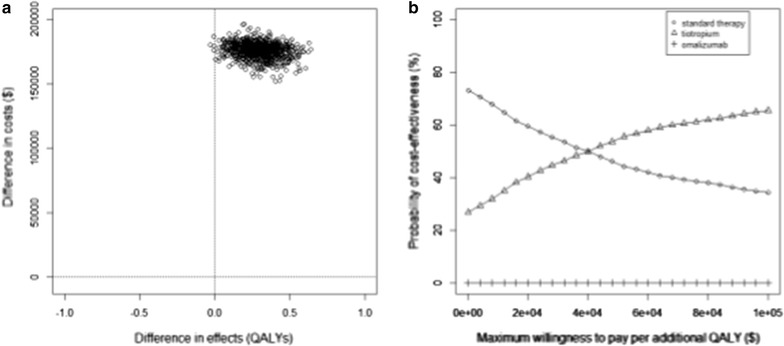
**a** Cost-effectiveness plane for add-on therapies with omalizumab versus tiotropium. **b** Cost-effectiveness acceptability curve for standard therapy, and add-on therapies with tiotropium and omalizumab

The results of the probabilistic analysis are presented in the Fig. 2. Figure 2a shows the cost-effectiveness plane for add-on therapy with omalizumab versus tiotropium. As seen in this figure, in the majority of the model runs, omalizumab had a higher effectiveness but also substantially higher costs compared with tiotropium (thereby lying mostly within the north-east quadrant of the cost-effectiveness plane). Figure 2b shows the cost-effectiveness acceptability curve. The probability that standard therapy would be the best option compared to the other two alternatives was 45% at WTP of $50,000/QALY, and 34% at WTP of $100,000/QALY. The corresponding probabilities for add-on therapy with tiotropium were 55% and 66%.

**Fig. 3:**
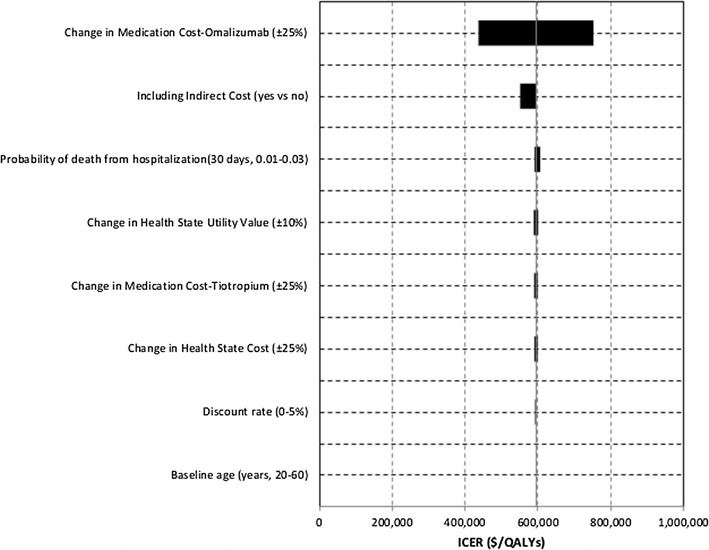
One-way sensitivity analysis: omalizumab versus tiotropium

Figure 3 shows the results of the sensitivity analyses. Changes in the cost of omalizumab had the greatest impact on outcomes. Reducing cost of omalizumab by 25% would decrease its ICER relative to tiotropium from the base case value to $436,944/QALY. In addition, increasing the cost of omalizumab by 25% would increase its base case ICER to $753,214/QALY relative to tiotropium. Changes in the other variables did not significantly alter the outcomes.

## Discussion

In this study, we compared the cost-effectiveness of three treatment strategies for uncontrolled allergic asthma. Our study indicated that add-on therapy with tiotropium was associated with an ICER of $34,478/QALY relative to standard therapy over a 10-year horizon. In contrast, add-on therapy with omalizumab had an ICER of $593,643/QALY relative to tiotropium. Therefore, the addition of tiotropium would be considered cost-effective compared to the other two alternatives at the conventionally used WTP of $50,000/QALY. Omalizumab showed the highest effectiveness both in terms of improvement in QALYs and reduction in the number of exacerbations, but this came with substantial incremental costs.

A number of studies have compared the costs and health outcomes of different treatments for uncontrolled asthma. Willson et al., whose model was adopted for this analysis, have reported on the cost-effectiveness of add-on therapy with tiotropium relative to standard therapy in the UK [10, 18]. Other studies have previously assessed the cost-effectiveness of add-on therapy with omalizumab versus standard therapy [3, 12–17]. Nonetheless, the cost-effectiveness comparison of an add-on therapy with tiotropium and omalizumab has not, to our knowledge, previously been assessed. This comparison is required for decision makers and stakeholders to base their treatment decision on an objective framework. The data reported in this study have therefore important clinical and policy implications.

There are some limitations in the present study. Studies of tiotropium in asthma have recruited both allergic and non-allergic asthma patients. By using evidence from such trials we assumed the same health benefits of tiotropium for allergic and non-allergic asthma patients. This assumption seems to be supported by the evidence in *a* post hoc analysis of the pivotal trials of tiotropium, that showed no difference between allergic versus non allergic subjects [26]. Unlike for tiotropium, we could not find any published transition probabilities for omalizumab across the seven health states used in this study [10, 18]. However, the lack of direct evidence should not preclude us from an objective evaluation of decisions that practitioners and policy makers currently face [27, 28]. To overcome this evidential gap, we used acceptable mathematical techniques to back-calculate these transition probabilities using the available evidence on treatment effect on exacerbation rates. Our calculations showed excellent internal validity, and the calculated ICER for omalizumab versus standard therapy is within the range of published ICERs [2, 3]. Ideally, future studies would directly compare the effect of tiotropium against omalizumab.

## Conclusions

In summary, this study provides evidence on the direct comparison of the health and economic outcomes associated with competing treatment options for uncontrolled allergic asthma patients who are not controlled with currently available inhaled therapies. Asthma outcomes have significantly improved over the past decades [29]. However, a significant minority of patients remains symptomatic despite conventional therapies and contributes a significant burden to the health care system. While the emerging novel therapies for asthma present exciting new treatment options, it is important to critically assess the cost-effectiveness of such treatments.

## Additional file

**Additional file 1: Appendix S1.** Estimating the transition probabilities for add-on therapy with omalizumab. **Appendix S2.** Estimating the medication costs per level of control.

## Abbreviations

ICER: incremental cost-effectiveness ratio
QALY: quality-adjusted life years
WTP: willingness to pay
ICS: inhaled corticosteroids
LABA: long-acting beta agonists
GINA: global initiative for asthma
CrI: credible interval
