# Supplementary material for: Cost-effectiveness of tiotropium versus omalizumab for uncontrolled allergic asthma in US
Source: Cost Eff Resour Alloc. 2018 Jan 30;16:3. doi: 10.1186/s12962-018-0089-8 (PMC5789632; doi:10.1186/s12962-018-0089-8)
Supplement: Supplementary file 1 — Additional file 1: Appendix S1. Estimating the transition probabilities for add-on therapy with omalizumab. Appendix S2. Estimating the medication costs per level of control. [file 12962_2018_89_MOESM1_ESM.docx]

Supplementary material table of contents

Appendix S1: Estimating the transition probabilities for add-on therapy with omalizumab

Appendix S2: Estimating the medication costs per level of control

Appendix S1: Estimating the transition probabilities for add-on therapy with omalizumab

To the best of our knowledge, there is no published study providing direct evidence on transition probabilities for omalizumab across the six alive health states in our model (i.e., controlled, partially controlled, uncontrolled, non-severe exacerbations, severe exacerbations without hospitalizations, and hospitalizations). Therefore, we used mathematical techniques to back-calculate these transition probabilities using the available indirect evidence.

**Indirect evidence:** We used a similar approach for our back-calculation for transition probabilities that was used in our recently published study (1). For this purpose, we used three pieces of evidence from the literature. 1) the relative rate (RR) of 0.489 for hospitalizations between omalizumab and standard therapy (2–4); 2) the RR of 0.617 for any type of exacerbations between omalizumab and standard therapy (2–4); and 3) the reported difference of 0.33 for change from baseline in Asthma Quality of Life Questionnaire (AQLQ) scores between omalizumab vs. standard therapy from a recent systematic review (5).

**Solving the transition matrix:**

We derived the transition probabilities for standard therapy, $tp_{ST}$, directly from literature (6,7). Assume the following matrix for $tp_{ST}$,

$tp_{ST}=\left[ \begin{matrix} tp_{1,1} & tp_{1,2} & tp_{1,3} & tp_{1,4} & tp_{1,5} & tp_{1,6} \\ tp_{2,1} & tp_{2,2} & tp_{2,3} & tp_{2,4} & tp_{2,5} & tp_{2,6} \\ tp_{3,1} & tp_{3,2} & tp_{3,3} & tp_{3,4} & tp_{3,5} & tp_{3,6} \\ tp_{4,1} & tp_{4,2} & tp_{4,3} & tp_{4,4} & tp_{4,5} & tp_{4,6} \\ tp_{5,1} & tp_{5,2} & tp_{5,3} & tp_{5,4} & tp_{5,5} & tp_{5,6} \\ tp_{6,1} & tp_{6,2} & tp_{6,3} & tp_{6,4} & tp_{6,5} & tp_{6,6} \end{matrix} \right]$,
where $tp_{1,1}$ to $tp_{6,6}$ show the transition probabilities for standard therapy across the six health states (as described in the main text).

We manipulated this matrix for standard therapy to arrive at transition matrix for omalizumab. To populate this matrix for omalizumab, we used three parameters that would capture the impact of omalizumab therapy on asthma control:

1) RR_1_ that represents the ratio in disease progression between omalizumab and standard therapy. Disease progression refers to the transitions from milder to more severe states (controlled, partially controlled, uncontrolled, and non-severe exacerbations).

2) RR_2_ for relative rate of transition from the afore-mentioned four health states to severe exacerbations without hospitalizations;

3) RR_3_ for relative rate of disease progression from the afore-mentioned four health states to hospitalizations.

We further assumed that the transition probabilities outgoing from severe exacerbations without hospitalizations and hospitalizations are not affected by the choice of treatments. Because we presumed once patients get exacerbations leading to an emergency room visit or a hospitalization, they receive a proper therapeutic (e.g., oral corticosteroids) or non-therapeutic management from a healthcare practitioner regardless of their underlying treatment. This assumption is supported by other published literature in this field (3,4).

Therefore, the transition probability for omalizumab, based on the transition probability for standard therapy and the afore-mentioned parameters is

$tp_{OM}=\left[ \begin{matrix} tp_{1,1} & tp_{1,2}*RR_{1} & tp_{1,3}*RR_{1} & tp_{1,4}*RR_{1} & tp_{1,5}*RR_{2} & tp_{1,6}*RR_{3} \\ tp_{2,1}*\frac{1}{RR_{1}} & tp_{2,2} & tp_{2,3}*RR_{1} & tp_{2,4}*RR_{1} & tp_{2,5}*RR_{2} & tp_{2,6}*RR_{3} \\ tp_{3,1}*\frac{1}{RR_{1}} & tp_{3,2}*\frac{1}{RR_{1}} & tp_{3,3} & tp_{3,4}*RR_{1} & tp_{3,5}*RR_{2} & tp_{3,6}*RR_{3} \\ tp_{4,1}*\frac{1}{RR_{1}} & tp_{4,2}*\frac{1}{RR_{1}} & tp_{4,3}*\frac{1}{RR_{1}} & tp_{4,4} & tp_{4,5}*RR_{2} & tp_{4,6}*RR_{3} \\ tp_{5,1} & tp_{5,2} & tp_{5,3} & tp_{5,4} & tp_{5,5} & tp_{5,6} \\ tp_{6,1} & tp_{6,2} & tp_{6,3} & tp_{6,4} & tp_{6,5} & tp_{6,6} \end{matrix} \right]$.

The final transition matrix was normalized such that the summation within reach row becomes 1.

Finally, we numerically estimated RR_1_, RR_2_, and RR_3_ using the three pieces of indirect evidence. The final values for RR_1_, RR_2_, and RR_3_ were 0.57, 0.66, and 0.49, respectively.

Appendix S2: Estimating the medication costs per level of control

To estimate the costs per level of control, we first obtained the average costs for being in no-exacerbation state (i.e., controlled, partially controlled, or uncontrolled) from Campbell et al. as $50/person-week (3). Second, we calculated the frequency of control states in the target population of our study through the stationary probabilities of the transition matrix. This resulted in the probability of being in controlled, partially controlled, and uncontrolled states as 0.25, 0.17, and 0.58, respectively. Third, we obtained the ratio of weekly costs across control states from a recent study by Sadatsafavi et al. (8). After adjusting to 2013 US dollars, these differential weekly costs were calculated as $0.73 for partially controlled vs. controlled, and $6.26 for uncontrolled vs. controlled (8–10).

Therefore, we had the following equation:
$0.25*x+0.17*\left( x+0.73 \right)+0.58*\left( x+6.26 \right)=50$,

where $x$ represents the costs of being in controlled state. Solving the equation for $x$, costs of being in controlled state becomes $46. Subsequently, the costs for partially controlled and controlled can be calculated as $47, and $53, respectively.

# References

1. Zafari Z, Lynd LD, FitzGerald JM, Sadatsafavi M. Economic and health effect of full adherence to controller therapy in adults with uncontrolled asthma: a simulation study. J Allergy Clin Immunol. 2014 Oct;134(4):908–915.e3.

2. Bousquet J, Cabrera P, Berkman N, Buhl R, Holgate S, Wenzel S, et al. The effect of treatment with omalizumab, an anti-IgE antibody, on asthma exacerbations and emergency medical visits in patients with severe persistent asthma. Allergy. 2005 Mar;60(3):302–8.

3. Campbell JD, Spackman DE, Sullivan SD. The costs and consequences of omalizumab in uncontrolled asthma from a USA payer perspective. Allergy. 2010 Sep;65(9):1141–8.

4. Zafari Z, Sadatsafavi M, Marra CA, Chen W, FitzGerald JM. Cost-Effectiveness of Bronchial Thermoplasty, Omalizumab, and Standard Therapy for Moderate-to-Severe Allergic Asthma. PloS One. 2016;11(1):e0146003.

5. Rodrigo GJ, Neffen H, Castro-Rodriguez JA. Efficacy and safety of subcutaneous omalizumab vs placebo as add-on therapy to corticosteroids for children and adults with asthma: A systematic review. CHEST J. 2011 Jan 1;139(1):28–35.

6. Willson J, Bateman ED, Pavord I, Lloyd A, Krivasi T, Esser D. Cost effectiveness of tiotropium in patients with asthma poorly controlled on inhaled glucocorticosteroids and long-acting β-agonists. Appl Health Econ Health Policy. 2014 Aug;12(4):447–59.

7. Willson J, Bateman ED, Pavord I, Lloyd A, Krivasi T, Esser D. Erratum to: Cost Effectiveness of Tiotropium in Patients with Asthma Poorly Controlled on Inhaled Glucocorticosteroids and Long-Acting β-Agonists. Appl Health Econ Health Policy. 2016 Feb;14(1):119–25.

8. Sadatsafavi M, Chen W, Tavakoli H, Rolf JD, Rousseau R, FitzGerald JM, et al. Saving in medical costs by achieving guideline-based asthma symptom control: a population-based study. Allergy. 2016 Mar 1;71(3):371–7.

9. 10-Year Currency Converter [Internet]. [cited 2014 Oct 16]. Available from: http://www.bankofcanada.ca/rates/exchange/10-year-converter/

10. US Inflation Calculator [Internet]. US Inflation Calculator. [cited 2014 Oct 16]. Available from: http://www.usinflationcalculator.com/
